# *N*,*N*-Bis(diphenyl­phosphan­yl)cyclo­butanamine

**DOI:** 10.1107/S1600536811027656

**Published:** 2011-07-16

**Authors:** Ilana Engelbrecht, Hendrik G. Visser, Andreas Roodt

**Affiliations:** a Department of Chemistry, University of the Free State, PO Box 339, Bloemfontein 9300, South Africa

## Abstract

In the title compound, C_28_H_27_NP_2_, the N atom adopts an almost planar geometry with the two P atoms and the C atom attached to it, with a distance of 0.066 (2) Å between the N atom and the C/P/P plane. The distorted trigonal–pyramidal geometry of the N atom is further illustrated by bond angles ranging between 115.22 (11) and 123.53 (8)°. Bond angles varying from 99.99 (9) to 108.07 (9) ° are indicative of the distorted pyramidal environment around the P atoms. An intra­molecular C—H⋯P hydrogen bond occurs. In the crystal, inter­molecular C—H⋯π inter­actions link the mol­ecules into a supra­molecular network.

## Related literature

For similar structures, see: Keat *et al.* (1981 ▶); Cotton *et al.* (1996 ▶); Fei *et al.* (2003 ▶); Cloete *et al.* (2008 ▶, 2009 ▶, 2010 ▶); Engelbrecht *et al.* (2010*a* ▶,*b* ▶). For diphosphinoamine (PNP) and other P-donor ligands, see: Muller *et al.* (2008 ▶); Purcell *et al.* (1995 ▶); Otto & Roodt (2001 ▶); Otto *et al.* (2005 ▶). For their use in catalytic olefin transformation reactions, see: Haumann *et al.* (2004 ▶); Crous *et al.* (2005 ▶); Booyens *et al.* (2007 ▶); Cloete *et al.* (2011 ▶); Ferreira *et al.* (2007 ▶). 
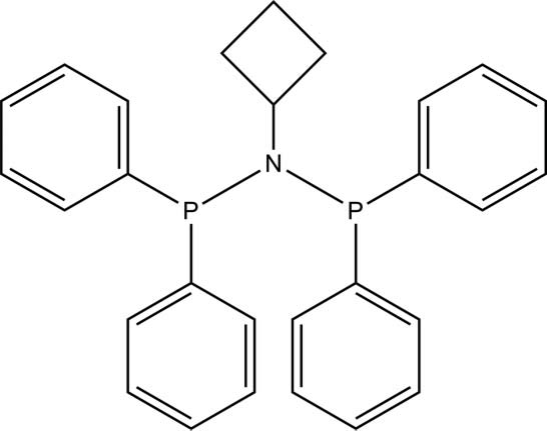


## Experimental

### 

#### Crystal data

C_28_H_27_NP_2_
*M* *_r_* = 439.45
Monoclinic, 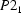

*a* = 9.414 (5) Å
*b* = 9.664 (5) Å
*c* = 12.644 (4) Å
β = 94.245 (5)°
*V* = 1147.2 (10) Å^3^
*Z* = 2
Mo *K*α radiation
μ = 0.21 mm^−1^
*T* = 100 K
0.32 × 0.12 × 0.04 mm

#### Data collection

Bruker X8 APEXII 4K KappaCCD diffractometer
Absorption correction: multi-scan (*SADABS*; Bruker, 2004 ▶) *T*_min_ = 0.937, *T*_max_ = 0.992
20748 measured reflections
3035 independent reflections
2930 reflections with *I* > 2σ(*I*)
*R*_int_ = 0.027

#### Refinement

*R*[*F* ^2^ > 2σ(*F* ^2^)] = 0.024
*wR*(*F* ^2^) = 0.064
*S* = 1.04
3035 reflections
281 parameters
1 restraint
H-atom parameters constrained
Δρ_max_ = 0.25 e Å^−3^
Δρ_min_ = −0.18 e Å^−3^

### 

Data collection: *APEX2* (Bruker, 2010 ▶); cell refinement: *SAINT-Plus* (Bruker, 2004 ▶); data reduction: *SAINT-Plus*; program(s) used to solve structure: *SHELXS97* (Sheldrick, 2008 ▶); program(s) used to refine structure: *SHELXL97* (Sheldrick, 2008 ▶); molecular graphics: *DIAMOND* (Brandenburg & Putz, 2005 ▶); software used to prepare material for publication: *WinGX* (Farrugia, 1999 ▶).

## Supplementary Material

Crystal structure: contains datablock(s) global, I. DOI: 10.1107/S1600536811027656/go2019sup1.cif

Structure factors: contains datablock(s) I. DOI: 10.1107/S1600536811027656/go2019Isup2.hkl

Additional supplementary materials:  crystallographic information; 3D view; checkCIF report

## Figures and Tables

**Table 1:** Hydrogen-bond geometry (Å, °) *Cg*1 and *Cg*2 are the centroids of the C11–C16 and C21–C26 rings, respectively.

*D*—H⋯*A*	*D*—H	H⋯*A*	*D*⋯*A*	*D*—H⋯*A*
C32—H32⋯P1	0.95	2.8	3.452 (2)	127
C43—H43⋯*Cg*1^i^	0.95	2.87	3.686 (7)	144
C44—H44⋯*Cg*2^ii^	0.95	2.81	3.614 (6)	143

Symmetry codes: (i) 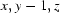; (ii) 
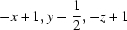
.

**Fig. 1.**
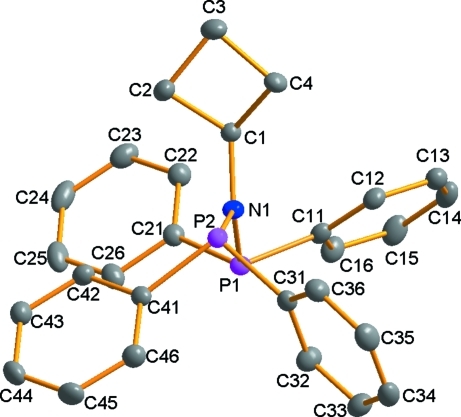
Molecular structure of the title compound. Displacement ellipsoids are drawn at the 50% probability level.

**Fig. 2.**
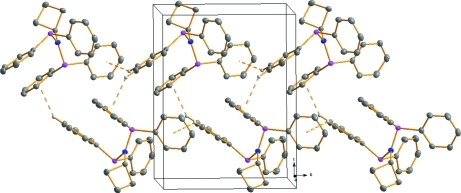
The crystal packing of the title compound, viewed along the a axis forming the three-dimensional framework. C—H···π interactions are shown as dashed lines. Displacement ellipsoids are drawn at the 50% probability level.

## supplementary crystallographic information

### Comment

Diphosphinoamine (PNP) and other P donor ligands (Muller *et al.*, 2008; Purcell *et al.*, 1995; Otto *et al.*,  2005; Otto & Roodt, 2001) with various substituents on both the P and N atoms form part of ongoing research in different catalytic olefin transformation reactions such as hydroformylation (Haumann *et al.*, 2004; Crous *et al.*, 2005), metathesis (Booyens *et al.*) methoxycarbonylation (Ferreira *et al.*, 2007) and tetramerization (Cloete *et al.*, 2011). In the title compound, C_29_H_29_NP_2_, Fig.1, all bond distances and angles fall within the range for similar complexes (Keat *et al.*, 1981; Cotton *et al.*, 1996; Fei *et al.*, 2003; Cloete *et al.*, 2008, 2009, 2010; Engelbrecht *et al.*, 2010*a*, 2010*b*).

The N(P_2_C) group is almost planar, with the central N displaced by -0.066 (2) Å from the P1—P2—C1 plane. The distorted trigonal-pyramidal geometry around the N atom is evident by the bond angles ranging between 115.22 (11) and 123.53 (8) °. The distorted triangular pyramidal geometry around the phosphorous atoms is indicated by C—P—C angles varying from 99.99 (9) - 108.07 (9) ° and N—P—C angles from 102.24 (8) - 108.07 (9) °. The phosphorous lone pairs are *trans* with respect to the N—C bond, therefore the title compound has a C_s_ conformer in the solid state. The crystal packing is stabilized by a C—H···P intramolecular hydrogen bond (Table 1) and intermolecular C—H···π interactions resulting in a three-dimensional network (Table 1, Figure 2).

### Experimental

Cyclobutylamine (0.010 mol, 854 µl) was dissolved in dichloromethane (30 ml) after which the solution was placed on an ice bath. Triethylamine (0.030 mol, 4.21 ml) was added to the solution while stirring. Chlorodiphenylphosphine (0.020 mol, 3.70 ml) was slowly added to the reaction mixture. The ice bath was removed after 1 h and the reaction mixture was allowed to stir at room temperature for a further 12 h. The dichloromethane was removed under reduced pressure. A mixture of hexane (20 ml) and toluene (2 ml) was added to the remaining white powder and was passed through a column containing neutral activated alumina (35 g). The solvent of the eluent was removed under reduced pressure and the white precipitate was collected. Single colourless crystals suitable for X-ray crystallography were obtained from recrystallization from methanol. (yield: 2.100 g, 48%)

### Refinement

The methine, methylene and aromatic H atoms were placed in geometrically idealized positions at C—H = 1.00, 0.99 and 0.95 Å, respectively and constrained to ride on their parent atoms, with *U*_iso_(H) = 1.2*U*_eq_(C). The highest peak is located 0.77 Å from C1 and the deepest hole is situated 0.55 Å from P1. In the absence of significant anomalous scattering effects, Friedel pairs have been merged.

### Crystal data

**Table:**

C_28_H_27_NP_2_	*F*(000) = 464
*M**_r_* = 439.45	*D*_x_ = 1.272 Mg m^−^^3^
Monoclinic, *P*2_1_	Mo *K*α radiation, λ = 0.71073 Å
Hall symbol: P 2yb	Cell parameters from 9952 reflections
*a* = 9.414 (5) Å	θ = 2.7–28.3°
*b* = 9.664 (5) Å	µ = 0.21 mm^−^^1^
*c* = 12.644 (4) Å	*T* = 100 K
β = 94.245 (5)°	Cuboid, colourless
*V* = 1147.2 (10) Å^3^	0.32 × 0.12 × 0.04 mm
*Z* = 2	

### Data collection

**Table:**

Bruker X8 APEXII 4K KappaCCD diffractometer	3035 independent reflections
Radiation source: fine-focus sealed tube	2930 reflections with *I* > 2σ(*I*)
graphite	*R*_int_ = 0.027
ω and φ scans	θ_max_ = 28.4°, θ_min_ = 2.2°
Absorption correction: multi-scan (*SADABS*; Bruker, 2004)	*h* = −12→12
*T*_min_ = 0.937, *T*_max_ = 0.992	*k* = −12→12
20748 measured reflections	*l* = −16→16

### Refinement

**Table:**

Refinement on *F*^2^	Primary atom site location: structure-invariant direct methods
Least-squares matrix: full	Secondary atom site location: difference Fourier map
*R*[*F*^2^ > 2σ(*F*^2^)] = 0.024	Hydrogen site location: inferred from neighbouring sites
*wR*(*F*^2^) = 0.064	H-atom parameters constrained
*S* = 1.04	*w* = 1/[σ^2^(*F*_o_^2^) + (0.0386*P*)^2^ + 0.1935*P*] where *P* = (*F*_o_^2^ + 2*F*_c_^2^)/3
3035 reflections	(Δ/σ)_max_ = 0.001
281 parameters	Δρ_max_ = 0.25 e Å^−^^3^
1 restraint	Δρ_min_ = −0.18 e Å^−^^3^

### Special details

**Table:**

Experimental. The intensity data were collected on a Bruker X8 ApexII 4 K Kappa CCD diffractometer using an exposure time of 40 s/frame. A total of 1709 frames were collected with a frame width of 0.5° covering up to θ = 28.39° with 99.9% completeness accomplished.
Geometry. All s.u.'s (except the s.u. in the dihedral angle between two l.s. planes) are estimated using the full covariance matrix. The cell s.u.'s are taken into account individually in the estimation of s.u.'s in distances, angles and torsion angles; correlations between s.u.'s in cell parameters are only used when they are defined by crystal symmetry. An approximate (isotropic) treatment of cell s.u.'s is used for estimating s.u.'s involving l.s. planes.
Refinement. Refinement of *F*^2^ against ALL reflections. The weighted *R*-factor *wR* and goodness of fit *S* are based on *F*^2^, conventional *R*-factors *R* are based on *F*, with *F* set to zero for negative *F*^2^. The threshold expression of *F*^2^ > 2σ(*F*^2^) is used only for calculating *R*-factors(gt) *etc*. and is not relevant to the choice of reflections for refinement. *R*-factors based on *F*^2^ are statistically about twice as large as those based on *F*, and *R*- factors based on ALL data will be even larger.

### Fractional atomic coordinates and isotropic or equivalent isotropic displacement parameters (Å^2^)

**Table:**

	*x*	*y*	*z*	*U*_iso_*/*U*_eq_	
C1	0.63564 (17)	0.73665 (19)	0.12318 (13)	0.0161 (3)	
H1	0.7182	0.7791	0.1657	0.019*	
C2	0.6829 (2)	0.5945 (2)	0.08102 (15)	0.0227 (4)	
H2A	0.7631	0.5515	0.1244	0.027*	
H2B	0.6039	0.528	0.0666	0.027*	
C3	0.72812 (19)	0.6710 (2)	−0.01884 (14)	0.0243 (4)	
H3A	0.8308	0.694	−0.016	0.029*	
H3B	0.697	0.6245	−0.0864	0.029*	
C4	0.63230 (18)	0.7937 (2)	0.00897 (13)	0.0202 (3)	
H4A	0.6781	0.8854	0.0035	0.024*	
H4B	0.5366	0.7926	−0.0291	0.024*	
C11	0.61059 (17)	0.96900 (19)	0.30492 (14)	0.0170 (3)	
C12	0.59621 (18)	1.04878 (19)	0.21255 (13)	0.0185 (3)	
H12	0.5452	1.0127	0.151	0.022*	
C13	0.65569 (17)	1.1803 (2)	0.20966 (14)	0.0207 (3)	
H13	0.6468	1.2325	0.1458	0.025*	
C14	0.72805 (19)	1.2358 (2)	0.29965 (16)	0.0238 (4)	
H14	0.7687	1.3257	0.2977	0.029*	
C15	0.7403 (2)	1.1586 (2)	0.39223 (15)	0.0259 (4)	
H15	0.7887	1.1963	0.4542	0.031*	
C16	0.68275 (19)	1.0269 (2)	0.39524 (14)	0.0223 (4)	
H16	0.6923	0.9752	0.4593	0.027*	
C21	0.67262 (18)	0.69892 (19)	0.37827 (13)	0.0177 (3)	
C22	0.81721 (19)	0.7224 (2)	0.36534 (13)	0.0204 (3)	
H22	0.845	0.7973	0.3228	0.024*	
C23	0.9200 (2)	0.6362 (2)	0.41469 (14)	0.0249 (4)	
H23	1.0179	0.6522	0.4053	0.03*	
C24	0.8808 (2)	0.5268 (2)	0.47773 (14)	0.0283 (4)	
H24	0.9515	0.4673	0.5103	0.034*	
C25	0.7388 (2)	0.5049 (2)	0.49284 (14)	0.0270 (4)	
H25	0.7118	0.431	0.5366	0.032*	
C26	0.6354 (2)	0.5909 (2)	0.44406 (14)	0.0216 (4)	
H26	0.5381	0.5759	0.4556	0.026*	
C31	0.21248 (16)	0.8079 (2)	0.13397 (12)	0.0165 (3)	
C32	0.17680 (19)	0.8748 (2)	0.22612 (14)	0.0227 (4)	
H32	0.232	0.8584	0.2909	0.027*	
C33	0.0619 (2)	0.9650 (2)	0.22457 (16)	0.0249 (4)	
H33	0.0406	1.0117	0.2876	0.03*	
C34	−0.02202 (19)	0.9870 (2)	0.13090 (16)	0.0241 (4)	
H34	−0.1016	1.0474	0.13	0.029*	
C35	0.0110 (2)	0.9203 (2)	0.03906 (15)	0.0251 (4)	
H35	−0.0464	0.9348	−0.025	0.03*	
C36	0.12781 (18)	0.8322 (2)	0.04019 (13)	0.0206 (4)	
H36	0.1504	0.7879	−0.0235	0.025*	
C41	0.30058 (17)	0.54508 (18)	0.21428 (12)	0.0156 (3)	
C42	0.40188 (18)	0.45205 (19)	0.25833 (14)	0.0188 (3)	
H42	0.4998	0.467	0.2484	0.023*	
C43	0.3623 (2)	0.33809 (19)	0.31634 (14)	0.0219 (4)	
H43	0.4331	0.2773	0.3472	0.026*	
C44	0.2191 (2)	0.3130 (2)	0.32932 (14)	0.0244 (4)	
H44	0.1914	0.2351	0.3687	0.029*	
C45	0.11719 (19)	0.4034 (2)	0.28400 (15)	0.0250 (4)	
H45	0.0191	0.3861	0.2915	0.03*	
C46	0.15723 (19)	0.5188 (2)	0.22774 (14)	0.0208 (3)	
H46	0.0864	0.5804	0.1982	0.025*	
N1	0.50881 (15)	0.74590 (16)	0.18514 (11)	0.0153 (3)	
P1	0.52394 (4)	0.79965 (6)	0.31465 (3)	0.01550 (9)	
P2	0.35464 (4)	0.67934 (6)	0.12377 (3)	0.01489 (9)	

### Atomic displacement parameters (Å^2^)

**Table:**

	*U*^11^	*U*^22^	*U*^33^	*U*^12^	*U*^13^	*U*^23^
C1	0.0156 (7)	0.0166 (7)	0.0163 (7)	0.0007 (6)	0.0022 (6)	−0.0001 (6)
C2	0.0231 (9)	0.0179 (8)	0.0279 (9)	0.0032 (7)	0.0082 (7)	−0.0003 (7)
C3	0.0249 (9)	0.0246 (9)	0.0245 (8)	−0.0014 (8)	0.0089 (7)	−0.0050 (8)
C4	0.0210 (8)	0.0228 (8)	0.0173 (7)	0.0008 (7)	0.0048 (6)	0.0025 (7)
C11	0.0123 (7)	0.0168 (8)	0.0217 (8)	0.0012 (6)	0.0006 (6)	−0.0022 (6)
C12	0.0183 (8)	0.0190 (8)	0.0181 (8)	0.0025 (6)	0.0006 (6)	−0.0033 (6)
C13	0.0183 (8)	0.0195 (8)	0.0247 (8)	0.0016 (7)	0.0034 (6)	0.0009 (7)
C14	0.0177 (8)	0.0173 (8)	0.0362 (10)	−0.0002 (7)	0.0007 (7)	−0.0030 (7)
C15	0.0217 (9)	0.0242 (10)	0.0301 (9)	0.0021 (7)	−0.0095 (7)	−0.0062 (8)
C16	0.0239 (9)	0.0222 (9)	0.0200 (8)	0.0037 (7)	−0.0037 (7)	−0.0016 (7)
C21	0.0202 (8)	0.0185 (8)	0.0142 (7)	0.0002 (7)	−0.0006 (6)	−0.0007 (6)
C22	0.0218 (8)	0.0218 (9)	0.0173 (8)	0.0007 (7)	−0.0007 (6)	−0.0004 (6)
C23	0.0221 (9)	0.0314 (10)	0.0206 (8)	0.0062 (7)	−0.0020 (7)	−0.0047 (7)
C24	0.0395 (11)	0.0248 (10)	0.0194 (8)	0.0130 (9)	−0.0074 (8)	−0.0038 (7)
C25	0.0448 (12)	0.0197 (9)	0.0156 (8)	0.0007 (8)	−0.0026 (7)	0.0019 (7)
C26	0.0278 (9)	0.0201 (8)	0.0166 (8)	−0.0038 (7)	−0.0006 (7)	−0.0003 (7)
C31	0.0139 (7)	0.0158 (7)	0.0197 (7)	−0.0015 (6)	0.0009 (6)	0.0011 (7)
C32	0.0213 (8)	0.0258 (9)	0.0206 (8)	0.0038 (7)	−0.0018 (7)	−0.0017 (7)
C33	0.0232 (9)	0.0237 (9)	0.0282 (9)	0.0033 (7)	0.0049 (7)	−0.0031 (7)
C34	0.0164 (8)	0.0206 (9)	0.0356 (10)	0.0017 (7)	0.0039 (7)	0.0055 (8)
C35	0.0207 (8)	0.0276 (10)	0.0262 (9)	−0.0003 (7)	−0.0038 (7)	0.0071 (8)
C36	0.0192 (8)	0.0239 (9)	0.0186 (8)	−0.0018 (6)	0.0001 (6)	0.0006 (6)
C41	0.0180 (8)	0.0145 (7)	0.0144 (7)	−0.0018 (6)	0.0017 (6)	−0.0027 (6)
C42	0.0164 (8)	0.0185 (8)	0.0216 (8)	0.0000 (6)	0.0025 (6)	−0.0016 (6)
C43	0.0242 (9)	0.0194 (9)	0.0223 (8)	0.0017 (7)	0.0026 (7)	0.0018 (7)
C44	0.0297 (9)	0.0185 (8)	0.0257 (8)	−0.0056 (8)	0.0074 (7)	0.0011 (7)
C45	0.0187 (8)	0.0247 (9)	0.0323 (10)	−0.0052 (7)	0.0073 (7)	−0.0018 (8)
C46	0.0181 (8)	0.0212 (8)	0.0231 (8)	−0.0006 (7)	0.0010 (6)	−0.0014 (7)
N1	0.0135 (6)	0.0178 (7)	0.0146 (6)	−0.0015 (5)	0.0012 (5)	−0.0021 (5)
P1	0.01451 (19)	0.0175 (2)	0.01444 (18)	−0.00069 (16)	0.00074 (14)	−0.00094 (16)
P2	0.01521 (19)	0.01517 (18)	0.01420 (18)	−0.00057 (16)	0.00048 (13)	−0.00147 (16)

### Geometric parameters (Å, °)

**Table:**

C1—N1	1.478 (2)	C24—C25	1.380 (3)
C1—C4	1.544 (2)	C24—H24	0.95
C1—C2	1.551 (2)	C25—C26	1.389 (3)
C1—H1	1	C25—H25	0.95
C2—C3	1.550 (3)	C26—H26	0.95
C2—H2A	0.99	C31—C32	1.395 (2)
C2—H2B	0.99	C31—C36	1.399 (2)
C3—C4	1.546 (3)	C31—P2	1.838 (2)
C3—H3A	0.99	C32—C33	1.388 (3)
C3—H3B	0.99	C32—H32	0.95
C4—H4A	0.99	C33—C34	1.390 (3)
C4—H4B	0.99	C33—H33	0.95
C11—C12	1.398 (2)	C34—C35	1.383 (3)
C11—C16	1.401 (2)	C34—H34	0.95
C11—P1	1.837 (2)	C35—C36	1.390 (3)
C12—C13	1.391 (3)	C35—H35	0.95
C12—H12	0.95	C36—H36	0.95
C13—C14	1.390 (3)	C41—C46	1.396 (2)
C13—H13	0.95	C41—C42	1.396 (2)
C14—C15	1.386 (3)	C41—P2	1.8271 (19)
C14—H14	0.95	C42—C43	1.389 (2)
C15—C16	1.384 (3)	C42—H42	0.95
C15—H15	0.95	C43—C44	1.391 (3)
C16—H16	0.95	C43—H43	0.95
C21—C26	1.396 (2)	C44—C45	1.389 (3)
C21—C22	1.401 (3)	C44—H44	0.95
C21—P1	1.8404 (19)	C45—C46	1.390 (3)
C22—C23	1.389 (3)	C45—H45	0.95
C22—H22	0.95	C46—H46	0.95
C23—C24	1.390 (3)	N1—P1	1.7138 (15)
C23—H23	0.95	N1—P2	1.7192 (16)
			
N1—C1—C4	120.84 (14)	C23—C24—H24	120.1
N1—C1—C2	119.96 (14)	C24—C25—C26	120.05 (18)
C4—C1—C2	88.95 (13)	C24—C25—H25	120
N1—C1—H1	108.5	C26—C25—H25	120
C4—C1—H1	108.5	C25—C26—C21	120.91 (18)
C2—C1—H1	108.5	C25—C26—H26	119.5
C3—C2—C1	87.72 (14)	C21—C26—H26	119.5
C3—C2—H2A	114	C32—C31—C36	118.23 (16)
C1—C2—H2A	114	C32—C31—P2	126.41 (13)
C3—C2—H2B	114	C36—C31—P2	115.22 (13)
C1—C2—H2B	114	C33—C32—C31	120.98 (17)
H2A—C2—H2B	111.2	C33—C32—H32	119.5
C4—C3—C2	88.91 (13)	C31—C32—H32	119.5
C4—C3—H3A	113.8	C32—C33—C34	120.06 (18)
C2—C3—H3A	113.8	C32—C33—H33	120
C4—C3—H3B	113.8	C34—C33—H33	120
C2—C3—H3B	113.8	C35—C34—C33	119.69 (17)
H3A—C3—H3B	111.1	C35—C34—H34	120.2
C1—C4—C3	88.10 (13)	C33—C34—H34	120.2
C1—C4—H4A	114	C34—C35—C36	120.21 (17)
C3—C4—H4A	114	C34—C35—H35	119.9
C1—C4—H4B	114	C36—C35—H35	119.9
C3—C4—H4B	114	C35—C36—C31	120.80 (17)
H4A—C4—H4B	111.2	C35—C36—H36	119.6
C12—C11—C16	118.15 (17)	C31—C36—H36	119.6
C12—C11—P1	122.11 (13)	C46—C41—C42	118.17 (16)
C16—C11—P1	119.48 (14)	C46—C41—P2	121.48 (13)
C13—C12—C11	120.80 (16)	C42—C41—P2	119.57 (13)
C13—C12—H12	119.6	C43—C42—C41	121.28 (16)
C11—C12—H12	119.6	C43—C42—H42	119.4
C14—C13—C12	120.33 (17)	C41—C42—H42	119.4
C14—C13—H13	119.8	C42—C43—C44	120.01 (17)
C12—C13—H13	119.8	C42—C43—H43	120
C15—C14—C13	119.28 (18)	C44—C43—H43	120
C15—C14—H14	120.4	C45—C44—C43	119.19 (17)
C13—C14—H14	120.4	C45—C44—H44	120.4
C16—C15—C14	120.65 (17)	C43—C44—H44	120.4
C16—C15—H15	119.7	C44—C45—C46	120.68 (17)
C14—C15—H15	119.7	C44—C45—H45	119.7
C15—C16—C11	120.78 (18)	C46—C45—H45	119.7
C15—C16—H16	119.6	C45—C46—C41	120.65 (17)
C11—C16—H16	119.6	C45—C46—H46	119.7
C26—C21—C22	118.63 (16)	C41—C46—H46	119.7
C26—C21—P1	116.11 (14)	C1—N1—P1	120.76 (11)
C22—C21—P1	125.26 (13)	C1—N1—P2	115.22 (11)
C23—C22—C21	120.07 (17)	P1—N1—P2	123.53 (8)
C23—C22—H22	120	N1—P1—C11	102.24 (8)
C21—C22—H22	120	N1—P1—C21	105.31 (8)
C22—C23—C24	120.50 (18)	C11—P1—C21	99.99 (9)
C22—C23—H23	119.7	N1—P2—C41	104.36 (7)
C24—C23—H23	119.8	N1—P2—C31	108.07 (9)
C25—C24—C23	119.79 (17)	C41—P2—C31	101.44 (8)
C25—C24—H24	120.1		
			
N1—C1—C2—C3	144.39 (15)	C42—C43—C44—C45	−0.2 (3)
C4—C1—C2—C3	18.85 (13)	C43—C44—C45—C46	−1.0 (3)
C1—C2—C3—C4	−18.83 (13)	C44—C45—C46—C41	1.0 (3)
N1—C1—C4—C3	−143.70 (15)	C42—C41—C46—C45	0.3 (3)
C2—C1—C4—C3	−18.90 (13)	P2—C41—C46—C45	170.16 (14)
C2—C3—C4—C1	18.91 (14)	C4—C1—N1—P1	−134.56 (15)
C16—C11—C12—C13	1.9 (2)	C2—C1—N1—P1	116.81 (15)
P1—C11—C12—C13	175.95 (13)	C4—C1—N1—P2	53.23 (19)
C11—C12—C13—C14	−1.4 (2)	C2—C1—N1—P2	−55.39 (19)
C12—C13—C14—C15	0.0 (3)	C1—N1—P1—C11	55.96 (14)
C13—C14—C15—C16	0.7 (3)	P2—N1—P1—C11	−132.50 (11)
C14—C15—C16—C11	−0.1 (3)	C1—N1—P1—C21	−48.12 (15)
C12—C11—C16—C15	−1.2 (3)	P2—N1—P1—C21	123.42 (11)
P1—C11—C16—C15	−175.36 (14)	C12—C11—P1—N1	26.64 (15)
C26—C21—C22—C23	2.1 (3)	C16—C11—P1—N1	−159.40 (14)
P1—C21—C22—C23	−177.74 (14)	C12—C11—P1—C21	134.85 (14)
C21—C22—C23—C24	−0.4 (3)	C16—C11—P1—C21	−51.19 (15)
C22—C23—C24—C25	−1.0 (3)	C26—C21—P1—N1	−104.69 (14)
C23—C24—C25—C26	0.8 (3)	C22—C21—P1—N1	75.13 (17)
C24—C25—C26—C21	0.9 (3)	C26—C21—P1—C11	149.56 (13)
C22—C21—C26—C25	−2.3 (3)	C22—C21—P1—C11	−30.62 (17)
P1—C21—C26—C25	177.53 (14)	C1—N1—P2—C41	121.40 (12)
C36—C31—C32—C33	−1.1 (3)	P1—N1—P2—C41	−50.57 (14)
P2—C31—C32—C33	−176.59 (15)	C1—N1—P2—C31	−131.22 (12)
C31—C32—C33—C34	1.8 (3)	P1—N1—P2—C31	56.81 (13)
C32—C33—C34—C35	−1.0 (3)	C46—C41—P2—N1	144.79 (14)
C33—C34—C35—C36	−0.3 (3)	C42—C41—P2—N1	−45.52 (15)
C34—C35—C36—C31	0.9 (3)	C46—C41—P2—C31	32.56 (16)
C32—C31—C36—C35	−0.2 (3)	C42—C41—P2—C31	−157.75 (13)
P2—C31—C36—C35	175.78 (14)	C32—C31—P2—N1	−50.48 (18)
C46—C41—C42—C43	−1.6 (2)	C36—C31—P2—N1	133.95 (13)
P2—C41—C42—C43	−171.62 (13)	C32—C31—P2—C41	58.92 (17)
C41—C42—C43—C44	1.6 (3)	C36—C31—P2—C41	−116.66 (14)

### Hydrogen-bond geometry (Å, °)

**Table:**

Cg1 and Cg2 are the centroids of the C11–C16 and C21–C26 rings, respectively.

**Table:**

*D*—H···*A*	*D*—H	H···*A*	*D*···*A*	*D*—H···*A*
C32—H32···P1	0.95	2.8	3.452 (2)	127.
C43—H43···Cg1^i^	0.95	2.87	3.686 (7)	144
C44—H44···Cg2^ii^	0.95	2.81	3.614 (6)	143.

Symmetry codes: (i) *x*, *y*−1, *z*; (ii) −*x*+1, *y*−1/2, −*z*+1.
